# How do Twitter users feel about telehealth? A mixed‐methods analysis of experiences, perceptions and expectations

**DOI:** 10.1111/hex.13927

**Published:** 2023-12-01

**Authors:** Hannah Sazon, Soraia de Camargo Catapan, Afshin Rahimi, Oliver J. Canfell, Jaimon Kelly

**Affiliations:** ^1^ School of Public Health The University of Queensland Brisbane Queensland Australia; ^2^ Centre for Online Health The University of Queensland Brisbane Queensland Australia; ^3^ Centre for Health Services Research The University of Queensland Brisbane Queensland Australia; ^4^ Queensland Digital Health Centre, Centre for Health Services Research, Faculty of Medicine The University of Queensland Brisbane Queensland Australia; ^5^ Digital Health Cooperative Research Centre Australian Government Sydney New South Wales Australia; ^6^ UQ Business School, Faculty of Business, Economics and Law The University of Queensland Brisbane Queensland Australia

**Keywords:** consumer satisfaction, Social Media, telehealth, telemedicine, user experience

## Abstract

**Background:**

Telehealth use has increased considerably in the last years and evidence suggests an overall positive sentiment towards telehealth. Twitter has a wide userbase and can enrich our understanding of telehealth use by users expressing their personal opinions in an unprompted way. This study aimed to explore Twitter users' experiences, perceptions and expectations about telehealth over the last 5 years.

**Methods:**

Mixed‐methods study with sequential complementary quantitative and qualitative phases was used for analysis stages comprising (1) a quantitative semiautomated analysis and (2) a qualitative research‐led thematic analysis. A machine learning model was used to establish the data set with relevant English language tweets from 1 September 2017 to 1 September 2022 relating to telehealth using predefined search words. Results were integrated at the end.

**Results:**

From the initial 237,671 downloaded tweets, 6469 had a relevancy score above 0.8 and were input into Leximancer and 595 were manually analysed. Experiences, perceptions and expectations were categorised into three domains: experience with telehealth consultation, telehealth changes over time and the purpose of the appointment. The most tweeted experience was expectations for telehealth consultation in comparison to in‐person consultations. Users mostly mentioned the hope that waiting times for the consultations to start to be less than in‐person, more telehealth appointments to be available and telehealth to be cheaper. Perceptions around the use of telehealth in relation to healthcare delivery changes brought about by the COVID‐19 pandemic were also expressed. General practitioners were mentioned six times more than other healthcare professionals.

**Conclusion/Implications:**

This study found that Twitter users expect telehealth services to be better, more affordable and more available than in‐person consultations. Users acknowledged the convenience of not having to travel for appointments and the challenges to adapt to telehealth.

**Patient or Public Contribution:**

An open data set with 237,671 tweets expressing users' opinions in an unprompted way was used as a source for telehealth service users, caregivers and members of the public experiences, perceptions and expectations of telehealth.

## 1 INTRODUCTION

Telehealth encompasses a variety of technology‐based and non‐face‐to‐face models of healthcare delivery, including telephone, video and computer‐based appointments.^1^ It allows increased access to health services in remote settings, particularly important during the COVID‐19 pandemic as non‐face‐to‐face models were necessitated to safely continue to provide healthcare.^1^, ^2^ Since this increase in the use of telehealth services, research on user experience of telehealth has increased considerably.^3^, ^4^, ^5^

Recent literature from 2021 to 2022 suggests an overall satisfaction towards telehealth.^2^, ^6^ Larger user satisfaction studies have been primarily survey‐based, while results from qualitative studies provide in‐depth insights relative to specific research settings and population groups. Also, relying on telehealth users' participation in research could introduce selection bias and a positive skew towards users' satisfaction and experience, potentially not reflecting the overall public opinion or real‐life experiences of those who are not digital literate to engage with the technology.^7^, ^8^ Furthermore, user satisfaction with telehealth has changed recently as it was new to many and often the only way to receive care during the pandemic; with its expansion and influx of its users, there are more people whose experiences have yet to be evaluated.^9^, ^10^

A way to gain an increased understanding of overall user experiences is to use Twitter as a data source.* While limited to those who have Internet access and are users of this specific Social Media platform, Twitter has over 230 million daily users^11^, ^12^ and provides a global sample of user‐generated content,^13^ freely expressed in an unprompted way,^10^, ^11^ through a 280 character maximum long post called a tweet.^14^ Previous studies have utilised Twitter to understand healthcare experiences and assist in identifying public health issues.^12^, ^15^, ^16^ Users primarily tweet their personal experiences when posting about health‐related topics,^16^ providing an important source of anecdotal evidence. The onset of the COVID‐19 pandemic observed a 13‐fold increase in telehealth‐related tweets.^10^ This increase can add to the current knowledge of telehealth.

Telehealth use has grown significantly,^17^, ^18^, ^19^ with a fourfold increase in telehealth use in the United States in 2020 compared to the previous year.^20^ Studies have analysed Twitter data over different time periods, from 2 months to 2 years, to examine opinions towards telehealth during the COVID‐19 pandemic, suggesting overall positive satisfaction (4) and social acceptance of telehealth (5). However, these studies were unable to illustrate the unique qualitative feelings and experiences of these users. If Twitter data can be analysed using machine learning, semiautomated software and qualitative methods pre, during and post COVID‐19, this would generate novel evidence of the unique and granular experiences of telehealth users on Twitter in a period of overall growth. Addressing this gap can help to characterise telehealth experiences, both positive and negative, with greater precision. This study aimed to explore the Twitter users' telehealth experiences, perceptions and expectations from September 2017 to September 2022.

## 2 METHODS

**Figure 1:**
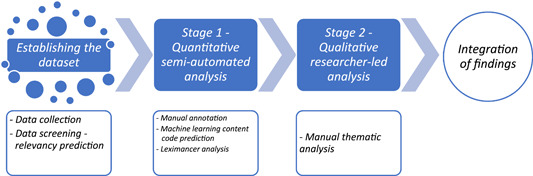
Mixed‐methods study design with sequential quantitative and qualitative phases.

A mixed‐methods study design with sequential complementary quantitative and qualitative phases was used for analysis.^21^ A machine learning model was used to establish the data set. Two analysis stages comprising (1) a quantitative semiautomated analysis and (2) a qualitative research‐led analysis^22^ were integrated at the end, when interpreting the findings. Figure 1 illustrates the study design stages.

### 2.1 Establishing the data set

An experienced author in computer science and Twitter data sets (A. R.) extracted data using a combination of keywords designed by telehealth researchers (J. K. and O. J. C.) to cover possible telehealth terminology adopted by English‐speaking Twitter users (Supporting Information: Material A). Tweets from 1 September 2017 to 1 September 2022 were included. Raw data was extracted to a Microsoft Excel spreadsheet and columns ‘date and time’ and ‘tweet’ were utilised for analysis.

Two authors (H. S. and S. d. C. C.) manually annotated 2000 tweets from the raw data, randomly 400 from each year, as relevant if they were related to telehealth experiences, perceptions and expectations (annotated as ‘1’) and not relevant (annotated as ‘0’). These annotations were used to train a machine learning model to predict the relevance of each tweet (A. R.) on a granular scale from 0 to 1, with closer to 1 as more relevant.

After an eye check on the predictions in decrescent order, a consensus between the authors established the relevance threshold above 0.8 due to the closer alignment of these tweets to the aim of the study. An Excel spreadsheet with tweets above the relevance threshold was produced for the next step – the data set.

### 2.2 Stage 1. Quantitative semiautomated analysis

The first author (H. S.) manually annotated the 500 highest relevant tweets to telehealth (0.9833–0.8833) using one word content code^22^ with a 5% random sample reviewed (S. d. C. C.). This data set was sufficient to train a classifier to predict the content code for each tweet in the data set using machine learning (A. R.). The content codes, their description, the number of tweets identified and an exemplar tweet are described in Supporting Information: Material B. This process helped researchers (H. S. and S. d. C. C.) refine their interpretation of the tweets, which can have multiple meanings not always captured by semiautomated analysis software. It also assisted researchers (H. S., S. d. C. C. and J. K.) in gaining familiarity with the entire data set, helping identify overarching categories described in Stage 2 (Qualitative researcher‐led analysis).

Leximancer is a desktop app that utilises machine learning to capture themes and concepts from a large, uploaded data set. The data set was uploaded into Leximancer Desktop (version 5.0) with a mix of default and personalised project settings (Supporting Information: Material C) to better analyse the data according to the research question. Concept maps were generated with circular heat‐mapped themes – red being the most frequent and interconnected theme, followed by orange, yellow, green and so forth.^23^, ^24^ A process of zooming in (theme size 75%, concept size 0%) and zooming out (theme size 40%, concept size 0%) was conducted to increase understanding of identified themes and concepts, with more or less granularity.^25^ This process with the one‐word content code prediction done previously in the manual annotation of the 500 highest relevant tweets allowed the identification of overarching categories.

Leximancer provided a synopsis table with descriptive statistics of each heat‐mapped circles quantifying text blocks as hits associated to each theme^23^ and showed associated concepts.^25^, ^26^, ^27^ Because Leximancer themes are named after the prevalence of text blocks and related concepts, they do not always represent accurately the overall meaning of the group of tweets they represent. We listed Leximancer theme bubbles (theme size 40%) and described them in what we called theme descriptions in a table. This table also included the number of hits for each Leximancer theme bubble and an example of an associated tweet to provide an overview of the overarching categories.

### 2.3 Stage 2: Qualitative researcher‐led analysis

The first author (H. S.) thematic analysed telehealth experiences, perceptions and expectations in an above 5% sample of the highest relevancy scores tweets (scored 0.89–0.9833).^22^ Half of this sample (50%) was blindly examined by an experienced qualitative researcher (S. d. C. C.) to ensure coding reflected consistent and appropriate interpretation. A peer‐debriefing meeting was conducted to reach consensus, and codes were quantified. The organisation of these codes resulted in overall categories and subcategories, guided by the concept maps resulting from the Leximancer analysis stage.

### 2.4 Integration of results from Stage 1 (quant) and Stage 2 (qual)

A final table was created to integrate the results by comparing and contrasting overarching categories, Leximancer themes and descriptions from the quantitative semiautomated analysis (Stage 1) and categories and subcategories from the qualitative researcher‐led analysis (Stage 2).

### 2.5 Ethical considerations

Exemption from ethics review was obtained from the University of Queensland's Research Ethics and Integrity (2023/HE000348). Users consent by accepting Twitter's terms of service and tweets are classified as open data.^28^ Data were stored on the University of Queensland's secure research storage server and will be destroyed in compliance with ethical use of Twitter data.^29^ To avoid further exposition of users' identity, usernames were replaced with ‘@mention', phone numbers were replaced with ‘X's and tweets were paraphrased using ChatGPT followed by a Google search to ensure the tweet could not be identified.

## 3 RESULTS

Raw data totalised 237,671 tweets extracted from 1 September 2017 to 1 September 2022, and 6469 tweets had a relevancy score above the threshold (0.81–0.9833), composing the analysed data set. Irrelevant tweets excluded totalised 231,102 with a relevancy score below the threshold (0–0.8). Table 1 gives examples of included and excluded tweets.

**Table 1:** Example of low‐scoring tweets excluded from the analysis and high‐scoring tweets included in the analysis.

Category	Score	Tweet^a^
Relevant tweets High Scoring (0.81–1.0)	0.856667	Just had an excellent telephone consultation with the GP – great communication from him – saved me time and it would've been the same in person.
	0.806667	Have a doc's appointment in 30 min and apparently it will be virtual or something?????
Irrelevant tweets Low Scoring (0–0.8)	0.45333	Just called to attempt to cancel my upcoming appt at this nightmare‐ish health clinic & OF COURSE no one answered and it took me to voicemail where phone messages go to die! Instead I cancelled online. So that's that! GOOD RIDDANCE!!!
	0.65333	Attn Students – reminder the Student Health Clinic is still open during the next semester. Call (XXX) XXX‐XXXX for phone screening and consultation.

Abbreviation: GP, general practitioner.

^a^ Paraphrased tweet from the ones posted by users.

### 3.1 Stage 1. Quantitative semiautomated analysis

**Figure 2:**
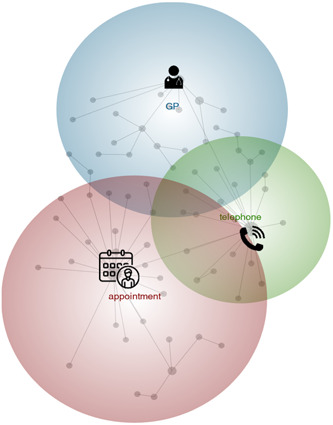
Leximancer concept map – theme size 75%, concept size 0% (icons added by the authors). GP, general practitioner.

A broader view of the Leximancer‐generated concept map (theme size 75%) with ‘appointment’ being the most important and interconnected theme, followed by general practitioner (GP) and telephone is illustrated in Figure 2.

**Figure 3:**
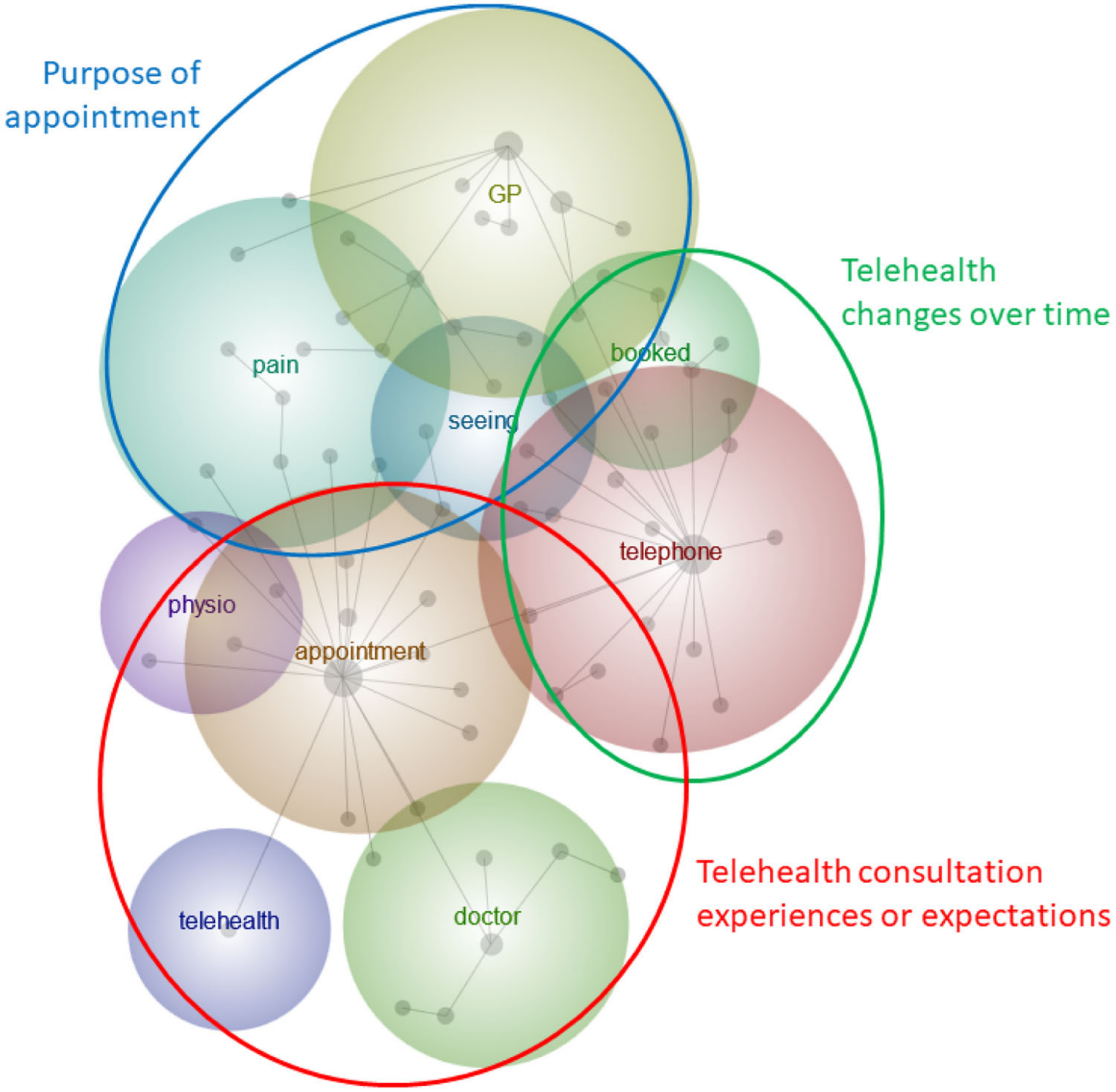
Leximancer concept map with overarching categories identified by the authors – theme size 40%, concept size 0%. GP, general practitioner.

By zooming out (theme size 40%), a more detailed concept map was provided (Figure 3). Themes bubbles were grouped into three overarching categories based on Figure 2 and contextual knowledge developed in previous manual annotations: (1) telehealth consultation experiences or expectations; (2) telehealth changes over time and (3) purpose of the appointment. Overarching categories were composed of 9 themes described into 8 themes descriptions encompassing over *n* = 23,193 text blocks (hits) summarised in Table 2.

**Table 2:** Summary of overarching categories, Leximancer themes and descriptions, number of hits and tweet examples from semiautomated analysis.

Overarching categories	Theme description	Leximancer Theme	Hits (*n* = 23,193)	Related Tweet example^a^
Telehealth consultation experiences or expectations	Expectation of telehealth consultations to be similar to in‐person	Appointment	7019	Another thing, now patients are actually waiting longer for a telephone appointment than they did for a face to face
	Waiting for healthcare professionals to start the appointment	Doctor	2397	I mean, I guess waiting on the phone is better than sitting in the waiting room for an hour
	Recommending telehealth	Telehealth	628	My doctor can do telephone visits or through video. Let me know bro!
	New experiences with telehealth	Physio	139	I've had bad back pain since the end of September and I have a physio appt next week – OVER THE PHONE. What use is that??
Telehealth changes over time	Response to the rapid uptake of telehealth due to COVID	Telephone	7456	My GP didn't directly say but mentioned we'll have a ‘new normal’. Haven't heard from her since.
		Seeing	148	Please explain to me why I am still struggling to see my local GP? After two years of living here, I have set foot in the office
	New healthcare delivery possibilities with telehealth	Booked	290	Since when do receptionists at the GP ask for a ‘short description’ of my health issue prior to booking a telephone appointment with my doctor?!
Purpose of appointment	Various purposes of appointments	GP	4965	Policy must be different for each GP practice. The surgery I go to examined both of my children last week without question after a brief telephone consultation.
	Use of telehealth for prescription refills	Pain	151	My routine telephone appointment tomorrow morning to discuss repeat medication. I am quite satisfied with a telephone appointment.

Abbreviation: GP, general practitioner.

^a^ Paraphrased tweet from the ones posted by users.

Under the overarching category ‘telehealth consultation experiences or expectations’, expectations of telehealth consultations to be similar to in‐person totalised *n* = 9416 hits, represented by ‘appointment’ and ‘doctor’ themes. The theme ‘appointment’ included expectations for telehealth to be similar or easier to book and be more available than in‐person appointments and the theme ‘doctor’ grouped tweets posted while users were waiting for healthcare professionals to start telehealth consultations. Still under ‘telehealth consultation experiences or expectations’, tweets reporting new experiences with different specialist services provided through telehealth were identified in the theme bubble ‘physio’ described as ‘new experiences with telehealth’ (Table 2). Tweets recommending telehealth appointments to other users were identified primarily in the theme bubble ‘telehealth’ described as ‘recommending telehealth’ (Table 2).

‘Telehealth changes over time’ overarching category grouped experiences with changes in healthcare delivery, summing *n* = 7894 hits. The theme bubbles ‘telephone’ and ‘seeing’ were grouped together and described as ‘response to the rapid uptake of telehealth due to COVID‐19’ (Table 2). For example, the telephone was used to triage patients before booking an appointment. Under the ‘booked’ theme bubble, new experiences as consequence of the pandemic‐related changes were described as ‘new healthcare delivery possibilities with telehealth’ (Table 2). Figure 3 shows ‘telephone’, ‘seeing’ and ‘booked’ theme bubbles overlap, representing the presence of tweets with interconnected and associated experiences.

‘GP’ and ‘pain’ theme bubbles were grouped under ‘purpose of the appointment’ overarching category with *n* = 5116 hits. While this overarching category has the least amount of hits, it is visualised as large theme bubbles in Figure 3 due to its connectedness to other themes in the concept map. Tweets under the ‘GP’ theme represented the ‘various purposes of telehealth appointments’, including reasons for seeking telehealth appointments, not exclusively with GPs. ‘Pain’ theme was described as ‘the use of telehealth for prescription refills’.

### 3.2 Stage 2. Qualitative researcher‐led analysis, integration of the results and interpretation of findings

Thematic analysis of 5% of the highest relevancy tweets identified 4 categories and 16 subcategories. Each category identified correlated to an overarching category provided by research insights over Leximancer concept maps (Stage 1). Table 3 displays the integration of results from the semiautomated (Stage 1) and researcher‐led analyses (Stage 2).

**Table 3:** Integration of semiautomated (Stage 1) and researcher‐led (Stage 2) results.

Stage 1. Quantitative semiautomated results (*n* = 23,193)			Stage 2. Qualitative researcher‐led results (*n* = 595)		
Overarching categories	Theme description	Leximancer theme (hits, %)	Category	Subcategory	Tweets count (%)
Telehealth consultation experiences or expectations	Recommending telehealth	Telehealth (628, 2.7%)	Recommending telehealth	Recommend others to ask for telehealth appointment	53 (8.9%)
	Expectation of telehealth to be similar to in‐person	Appointment hits (7019, 30.3%)	Telehealth consultation experiences or expectations	Length of telehealth consultation	13 (2.2%)
				‘Getting ready’ for telehealth consultation	11 (1.8%)
				Interruptions and lack of privacy during telehealth consultations	6 (1%)
				Availability of telehealth consultation	46 (7.7%)
				Comment on cost of telehealth consultation	10 (1.7%)
	Waiting for healthcare professionals to start the appointment	Doctor (2397, 10.3%)		Waiting for a telehealth consultation to start, doctors are late or not attending telehealth consultation	91 (15.3%)
	New experiences with telehealth	Physio (139, 0.6%)		Patient's first‐time using telehealth or expectations with future telehealth appt	31 (5.2%)
				General comments on telehealth experience – positive (14), negative (21), neutral (29)	64 (10.8%)
Telehealth changes over time	Response to the rapid uptake of telehealth due to COVID	Telephone (7456, 32.1%)	Changes due to the rapid uptake of telehealth consultation	Questioning when things will ‘go back to normal’ or start using face to face again	17 (2.9%)
		Seeing (148, 0.6%)			
	New healthcare delivery possibilities with telehealth	Booked (290, 1.3%)		Changes in workspace, workflows and/or healthcare delivery	49 (8.2%)
Purpose of appointment	Purpose of appointment	GP (4965, 21.4%)	Purpose or specialties of telehealth consultation	GPs	175 (29.4%)
				Mental health	8 (1.3%)
				Specialists	6 (1%)
				Physios	2 (0.3%)
	Use of telehealth for prescription refills	Pain (151, 0.7%)		Prescription refills	13 (2.2%)

Abbreviation: GP, general practitioner.

Identified subcategories in the researcher‐led analysis provided a deeper understanding of the results from the semiautomated analysis. For example, from the one theme description identified in the semiautomated analysis – ‘expectation of telehealth consultations to be similar as in‐person’ – five subcategories were identified. Tweets in these subcategories expressed users' expectations from a telehealth consultation to have the same duration, to be more available and to be cheaper than an in‐person appointment.

The subcategory ‘waiting for a telehealth consultation to start, doctors are late or not attending telehealth consultation’ was the most tweeted experience (*n* = 91 tweets) in the researcher‐led analysis and also had a high number of hits in the semiautomated analysis (*n* = 2397 hits). A tweet identified under this subcategory expressed the frustration and hope that waiting times for telehealth to start would be shorter or inexistent: ‘I downloaded Zoom for my online doctor's appointment and had to wait for a long time. As the waiting continued, I started to believe that the medical professionals were trying to replicate the in‐person experience because the wait time was the same!’.

The subcategory ‘general comments on telehealth experience’ was further divided into positive (14 tweets), negative (21 tweets) or neutral, where neither positive nor negative sentiment could be interpreted (29 tweets). An example of a positive tweet, which alludes to the convenience of telehealth, is: ‘This year, I had my yearly rheumatology appointment via telephone. It was convenient as I didn't have to leave work, make up for lost time, drive, or look for a parking spot. […] I could easily get used to this’. On the contrary, negative experiences raised challenges of adapting to this new way of communicating or difficulties with the technology: ‘During my initial online medical consultation today, it was difficult to handle turn‐taking. Due to a lagging issue, it seemed like I was frequently interrupting my doctor. There is a lot to consider and think about regarding human interaction in this situation’. Neutral experiences expressed telehealth participation without a clear opinion: ‘I am currently participating in a medical consultation via telehealth over the phone’.

A tweet under the subcategory ‘changes in workspace, workflows and/or health care delivery’ illustrates that telehealth provision involves background work that patients might often be unaware of: ‘Video consultations with patients take up just as much time as face‐to‐face consultations for GPs, and more than half of them end up requiring a face‐to‐face anyway. In fact, they take up even more time, and that's not even considering the significant expense of establishing them. The only way they make economic sense is if GPs cherry pick straightforward consultations, which is what some businesses do’.

Similar to the semiautomated analysis, telehealth consultations were most commonly used to see a GP in the research‐led analysis (*n* = 175 tweets, six times more than any other mentioned speciality) as seen in Table 3. Other specialties identified in the research‐led analysis were mental health – inclusive of psychiatrists and psychologists – and physiotherapists. The Leximancer identified theme ‘pain’ with theme descriptions ‘use of telehealth for prescription refills’ was further explored in the research‐led analysis as the use of telehealth not only to get prescription refills – often for chronic pain – but also as a convenient alternative when in‐person appointments are difficult to book: ‘Is it possible for someone to provide me with a birth control prescription via phone? Scheduling a doctor's appointment while working is just a pain [not ideal]’.

## 4 DISCUSSION

The aim of this study was to explore Twitter users' experiences, perceptions and expectations about telehealth in the 5 years surrounding the pandemic, from 1 September 2017 to 1 September 2022. The primary finding suggests that telehealth experience and expectations among Twitter users may differ from overall satisfaction previously reported.^4^, ^5^, ^7^, ^8^ Users have a general expectation for telehealth to be cheaper, more widely available and easier to book, with minimal delays to start, and with similar length to an in‐person consult. Care delivered via telehealth have background work that users seem to be unaware of and negative experiences on the challenges to adapt to this new modality of care delivery are also reported. The healthcare professional delivering telehealth primarily mentioned were GPs, who were also commonly recommended by users. Convenience of telehealth was also highlighted.

The perception that waiting times should be alleviated and bookings should be broadly available indicates that users might not have a complete grasp of what telehealth requires in terms of set‐up time, resources, administrative support and changes to usual workflows.^30^ There seems to be a disconnect between the virtual and real‐world healthcare experience. A phenomenon called the ‘online disinhibition effect’, where people are more critical on the internet and forget that there is a real person on the other end of the service, may help to understand these misconceptions.^31^ This idea is also represented in the sarcastic, ironic and emotionally charged tweets expressing frustration with doctor delays and the struggle encountered when trying to book a telehealth appointment. This finding further supports the need to combine methods and use more than automated software or AI models when analysing social media data.

Primary care GPs and mental health providers have been high users of telehealth – particularly during and after the COVID‐19 pandemic – in countries like Australia where these services have been funded by the public sector since the pandemic.^9^, ^32^, ^33^ This in part may explain the frequent GP mentions in our study data, but not psychiatrists/mental health providers and specialists in general. A possible explanation could be that while around 30% of total mental health consultations occur via telehealth in the United States and Australia, the number of users is not significant when compared to GP users.^9^, ^32^ Further analyses are necessary to understand users' experiences and expectations on mental health services via telehealth.

Physiotherapy was a speciality that emerged from our results and reflected people's new experiences with telehealth. Telehealth has been a convenient and effective way for people living in rural and remote areas to access allied healthcare services way before the pandemic.^34^ However, possibly due to the physical nature of this practice,^35^ or its low uptake before the pandemic,^36^ or still the potential efforts to overcome the digital divide or funding constrains developed in response to the COVID‐19 pandemic changes,^34^, ^37^ there was a growing number of people accessing physiotherapy via telehealth, hence our results. This showcases the innovation and expansion of telehealth and its potential to be integrated to optimise models of care.

Recent studies examined users' experiences and satisfaction towards telehealth during COVID‐19 using Twitter, while our study looked at a wider timeframe to include more experiences.^4^, ^5^ Similar to our results, these studies report users' questioning the return of healthcare delivery to its prepandemic model. These studies also echoed overall positive satisfaction and experiences with telehealth reported in existing literature,^4^, ^5^ suggesting social acceptance of telehealth. From our results, we could not assume an overall positive satisfaction or experience towards telehealth. The number of tweets with mixed or dual meanings relying on understanding the context for interpretation justifies the need for a mixed‐methods approach to provide a broader understanding of telehealth experiences that recent qualitative studies have not captured.^4^, ^5^, ^8^ Further, negative experiences with telehealth consultations and hurdles to adjusting to this modality of care have also been demonstrated in our results.

As technology continues to develop and telehealth becomes normalised, users should be educated about telehealth to prevent frustrations and align expectations to the existing capacity of service delivery. In turn, this understanding could assist first‐time users of telehealth and alleviate some of their concerns and anxieties. Future studies involving subgroup focused data and healthcare specialties could provide increased understanding of targeted user experiences and more nuanced improvements in healthcare delivery. Focused interventions for increasing telehealth engagement and the integration of telehealth as part of the health service can promote positive development.

### 4.1 Strengths and limitations

The inclusion of the human‐led components to machine learning and a semiautomated analysis provided a deep understanding of the context and interpretation of experiences with multiple meanings, resulting in novel findings. Also, the comprehensive search strategy resulted in a large raw data set. Limitations to using quantitative methods to analyse subjective data remain. Additional efforts to manually annotate larger samples of the data set could have improved the diversity of the findings. Another limitation was the attributes of the data source. Data obtained from online platforms is likely to exclude the views of people with lower digital literacy. Further, differences in popularity of social media platforms across countries should also be acknowledged. Additionally, this study only included tweets in English, excluding non‐English speaking users' experiences limiting the study's coverage. Furthermore, the socioeconomic class of telehealth users vary among countries which may indicate that telehealth users may still be unrepresented in the used data set. Overall, care should be taken in generalising these findings as they might not represent telehealth perspectives and experiences of the whole population.

## 5 CONCLUSION

The increase in both telehealth service delivery and mention of telehealth on Twitter showcased a variety of experiences, perceptions and expectations of telehealth of Twitter users. Mixed‐methods analysis assisted a more nuanced understanding beyond overall telehealth user satisfaction. While users discussed the general changes caused by the pandemic, their expectations for telehealth to provide a better experience, more affordable and available when compared to in‐person consultations were commonly expressed. Users acknowledged the convenience and the challenges to adapt to telehealth. These findings suggest the potential to improve telehealth delivery experience by raising awareness of its processes and addressing users' expectations. Further studies involving larger samples and multiple methods can substantiate these findings.

## AUTHOR CONTRIBUTIONS

*Project conception*: Jaimon Kelly. *Experimental design*: Hannah Sazon, Soraia de Camargo Catapan, Afshin Rahimi, Oliver J. Canfell and Jaimon Kelly. *Data collection*: Hannah Sazon, Soraia de Camargo Catapan, Afshin Rahimi, Oliver J. Canfell and Jaimon Kelly. *Data analysis*: Hannah Sazon, Soraia de Camargo Catapan and Jaimon Kelly. *Writing and editing the paper*: Hannah Sazon, Soraia de Camargo Catapan and Jaimon Kelly. *Submission*: Hannah Sazon and Soraia de Camargo Catapan.

## CONFLICT OF INTEREST STATEMENT

The authors declare no conflict of interest.

## ETHICS STATEMENT

Exemption from ethics review was obtained from the University of Queensland Research Ethics and Integrity (2023/HE000348). Users consent by accepting Twitter's terms of service stating tweets will be made available to third parties and therefore classifies as open data.^28^

## Supporting information

Supporting information.

Supporting information.

Supporting information.

## Data Availability

The data that support the findings of this study are available from the corresponding author upon reasonable request. Raw data or the data set used for analysis are available on request.

## Footnotes

* This study was conducted before Twitter was renamed to ‘X’ (July/2023). The change of name does not affect our results and we will refer to ‘X’ as Twitter in this paper.
