# Supplementary material for: How do Twitter users feel about telehealth? A mixed‐methods analysis of experiences, perceptions and expectations
Source: Health Expect. 2023 Dec 1;27(1):e13927. doi: 10.1111/hex.13927 (PMC10726278; doi:10.1111/hex.13927)
Supplement: Supplementary file 1 — Supporting information. [file HEX-27-e13927-s002.docx]

## **Supplementary Material A. Keywords used to establish Twitter database**

(telephone consult) OR (telephone consultation) OR (telephone counselling) OR (telephone appt) OR (phone appt) OR (phone consult) OR (phone consultation) OR (phone counselling) OR (video counselling) OR (video consult) OR (video consultation) OR (video appt) OR (videoconference appt) OR (video conference appt) OR (telehealth) OR (tele–health) OR (tele health) OR (teleconsult) OR (teleconsultation) OR (tele-consult) OR (tele consult) OR (tele-consultation) OR (tele consultation) OR (tele counselling) OR (telemedicine) OR (tele-medicine) OR (tele medicine) OR (tele appt) OR (tele-health appt) OR (virtual consult) OR (virtual consultation) OR (virtual counselling) OR (virtual appt) OR (virtual appointment) OR (online consult) OR (online consultation) OR (online counselling) OR (online appt) OR (econsult) OR (econsult) OR (econsultation) OR (e-consultation) OR (ecounselling) OR (e-counselling) OR (electronic consult) OR (electronic consultation) OR (electronic counselling) OR (electronic appt) OR (mobile consult) OR (mobile consultation) OR (mobile consultation) OR (mobile counselling) OR (mobile appt) OR (cell appt) OR (cellphone appt) OR (cell counselling) OR (cellphone counselling) OR (face to face consultation) OR

(face to face consult) OR (phone doctor) OR (video doctor)
