# Supplementary material for: How do Twitter users feel about telehealth? A mixed‐methods analysis of experiences, perceptions and expectations
Source: Health Expect. 2023 Dec 1;27(1):e13927. doi: 10.1111/hex.13927 (PMC10726278; doi:10.1111/hex.13927)
Supplement: Supplementary file 2 — Supporting information. [file HEX-27-e13927-s003.docx]

**Supplementary Material B**. Manual one-word content codes, code description, tweet exemplars and the total number of tweets identified by machine learning for each code from the final dataset.

| Content code | Code description | Tweet Exemplar* | | # of tweets identified |
| --- | --- | --- | --- | --- |
| Training | Train health professionals for providing telehealth | What does GP training involve these days? Is it about teaching them how to conduct consultations over the phone? | 1 | |
| Work | Workplace setting regards to use of telehealth | My entire clinic is conducting appointments via telehealth today. It would be great if they allowed me to work from home. | 3 | |
| Preference | Expresses preference for a health care service | As a patient, I would rather have a telephone consultation than expose myself to potential illness by sitting in a waiting room with other sick individuals. | 4 | |
| Ease | Ease of engaging with telehealth | I phoned my doctor's office to schedule a telephone appointment. The appointment was arranged for five minutes later, and I spoke with the doctor, who booked me for a blood test next week. Everything was completed in 30 minutes! Bless the NHS. | 6 | |
| Resistance | Expresses resistance to healthcare services | My GP only conducts telephone consultations, which is why I never contact them. It's not because I'm healthy, but because it involves making a phone call. | 6 | |
| Technology | Expresses use of a technology | I have a virtual appointment with my psychiatrist in 15 minutes, but I haven't received any instructions from the doctor regarding where to go or what to click, so I guess I'm the psychiatrist now. | 7 | |
| Transition | Transition from face-to-face services to telehealth | As of today, ALL GP appointments are now being done over a telephone consultation only. These are crazy times. | 9 | |
| Integration | Integration of telehealth and in person or other healthcare services | The ability to refer patients via telehealth makes the argument against it invalid. I have a telehealth consultation scheduled with a doctor I met for the first time last week to get a prescription for Clexane. He has no knowledge of my medical history except for the fact that I came to his office on crutches and with a leg brace. | 9 | |
| Access | Telehealth affecting access to healthcare | A Deaf patient received a letter cancelling a face-to-face appointment for a phone call follow-up, not a very good idea. | 10 | |
| Absent | Doctor does not attend telehealth appointment or patient expresses that doctor is missing from scheduled appointment | I was even stood up by my doctor during our virtual appointment. | 14 | |
| New | New/first time experience with telehealth | I have scheduled my mother's first telehealth appointment today, and I am curious to see how it goes. | 21 | |
| Feeling | Expresses emotions/feelings about telehealth | Oh my goodness, everyone! I made it through my first virtual doctor appointment through telehealth! I was really anxious, and it was over in just 45 seconds. I really dislike any kind of video chat or call, but I made it through. And I'm still here! | 66 | |
| Expectation | Expectation from telehealth services, i.e., cost, waiting time, appointment wait times | If I am able to have a cardiology appointment over the phone, then having a GP appointment on camera should be a piece of cake. | 145 | |
| Recommendation | Recommendation for telehealth or ask provider if they offer telehealth services | Inquire with your GP office regarding the possibility of telephone consultations as an alternative option for appointments. | 198 | |
| Experience | Expresses their experience with telehealth | I had a phone consultation with a GP working outside of normal hours, and they diagnosed me with IBS. However, two days later, I underwent surgery to remove my appendix. | 202 | |
| Late | Doctor is late to telehealth appointment | How is my dr late to a virtual appointment | 406 | |
| Use | Shows that telehealth is being used | Were you able to get a dr’s telephone appointment? By the way, I find it unacceptable that the doctor won't come to your location to see you and your daughter. | 5367 | |

*Paraphrased tweet posted by user
