# Supplementary material for: How do Twitter users feel about telehealth? A mixed‐methods analysis of experiences, perceptions and expectations
Source: Health Expect. 2023 Dec 1;27(1):e13927. doi: 10.1111/hex.13927 (PMC10726278; doi:10.1111/hex.13927)
Supplement: Supplementary file 3 — Supporting information. [file HEX-27-e13927-s001.docx]

**Supplementary Material C**. Leximancer project settings – same for both concept maps except whereas noted in steps 3 and 8. Table adapted from Haynes et al [25].

| Step‐by‐step process of analysis in Leximancer | |
| --- | --- |
| Step | Process Options (Our Command in Bold) |
| 1. Select documents | Select .csv with pre-organised tags |
| 2. Text processing settings | Sentences per block: 1,**2** (normal),3,4,5,6,10,20,100  Prose test threshold: **0** (default),1,2,3,4,5  Duplicate text sensitivity: **Off**, Auto, 1,2,3,4,5,6,7,8  Identify name‐like concepts: **Yes**/No  Break at paragraph: **On**/Off  Auto‐paragraphing: **On**/Off  Merge word variants: **On**/Off  Tags: **File**, **Folder**, Dialogue  Stop List: English language list  Remove: to  Add: thought |
| 3. Concept seeds setting | Automatically identify concepts: **On**/Off  Total number of concepts: **Automatic**, 10,20,30,40,50,60,70,80,90,100,110,120,130,140,150,160,170,180,190,  200,250,300,350,400,450,500,750,1000  Percentage of name‐like concepts: **Automatic**,10,20,30,40,50,60,70,80,90,100.  Mark Tags as Attributes: Off |
| Generate concept seeds | |
| 4. Edit concept seeds | Auto concepts or tags: No change  Concepts removed: ‘amp’, ‘due’, ‘having’, ‘ve’, ‘via’, ‘sure’, ‘to’, ‘long’, ‘least’, ‘doing’, ‘able’, ‘things’, ‘NHS’, and words with non-English characters and symbols  Concepts merged: ‘appointment, appt’ ‘doctor, dr’ ‘people, person’ ‘tele-health, telehealth’ ‘TELEPHONE, telephone, phone’ ‘tell, told’ ‘prescription, medication’ ‘people, person’  User defined **concepts** or tags: ‘therapists, therapy, psychiatrist’ and **merged** |
| 5. Thesaurus settings (concept learning) | Learn thesaurus from source documents: **Yes**/No  Learn once: On/**Off**  Concept generality: 1,2,3,4,5,6,7,8,**9**(default),10,11,12,13,14,15,16,17,18,19,20,21.  Learn from tags: On/**Off**  Learning type: **Normal**/Supervised  Sampling: **Automatic**,1,2,3,4,5,6,7,8,9,10  Sentiment lens: On/**Off**  Number to discover: **Off**, 10,20,30,40,50,60,70,80,90,100,110,120,130,140,150,160,170,180,190,200,250,300, 350,400,450,500,750,1000  Themed discovery, **concepts in any**/all/each  Only discover name‐like concepts: On/**Off** |
| 6. Topic Guide Settings | Topic Guide Enabled: **Yes** (default), No  Topic Guide PowerPoint Generation: Yes, **No** (default)  Minimum Topic Percentage: 15 |
| Generate thesaurus | |
| 7. Compound concepts | Choose any from list: ‘tele’ and ‘health’ |
| 8. Concept coding | Selected Mapping Concepts: All names/**all concepts**/**All discovered names**/All discovered concepts/All user names/All user concepts.  Required concepts: From list as stated above—None selected  Kill concepts: None  Options: All default settings. |
| 9. Project output settings | Map type: social network/**topical network**  Default theme size percentage: 10,15,20,25,30, 33, 35,40,45,50,55,**60** (default), 65  Map width: Auto  Map height: Auto  Generate concept map |
